# COVID-19 Echo Chambers: Examining the Impact of Conservative and Liberal News Sources on Risk Perception and Response

**DOI:** 10.1089/hs.2020.0176

**Published:** 2021-02-18

**Authors:** Kenneth A. Lachlan, Emily Hutter, Christine Gilbert

**Affiliations:** Kenneth A. Lachlan, PhD, is a Professor and Department Head; and Emily Hutter, MA, and Christine Gilbert, MA, are Graduate Assistants; all in the Department of Communication, University of Connecticut, Storrs, CT.

**Keywords:** COVID-19, Public health management/response, Health communication, News bias, New media, Risk perception

## Abstract

The coronavirus disease 2019 (COVID-19) pandemic has created substantial challenges for public health officials who must communicate pandemic-related risks and recommendations to the public. Their efforts have been further hampered by the politicization of the pandemic, including media outlets that question the seriousness and necessity of protective actions. The availability of highly politicized news from online platforms has led to concerns about the notion of “echo chambers,” whereby users are exposed only to information that conforms to and reinforces their existing beliefs. Using a sample of 5,000 US residents, we explored their information-seeking tendencies, reliance on conservative and liberal online media, risk perceptions, and mitigation behaviors. The results of our study suggest that risk perceptions may vary across preferences for conservative or liberal bias; however, our results do not support differences in the mitigation behavior across patterns of media use. Further, our findings do not support the notion of echo chambers, but rather suggest that people with lower information-seeking behavior may be more strongly influenced by politicized COVID-19 news. Risk estimates converge at higher levels of information seeking, suggesting that high information seekers consume news from sources across the political spectrum. These results are discussed in terms of their theoretical implications for the study of online echo chambers and their practical implications for public health officials and emergency managers.

## Introduction

The coronavirus disease 2019 (COVID-19) pandemic has created substantial challenges for public health officials and those responsible for informing the public about risks and providing recommendations to prevent further spread of the disease. As our knowledge of the virus has improved and infection and fatality rates change, the public has experienced a steady stream of information from online news sources that have varying degrees of credibility. Public health recommendations have become politicized, further hampering communication efforts, as the severity of the pandemic and need for behavioral interventions have been downplayed by media outlets with conservative biases. While risk communication research generally supports the notion that more informed audiences make better health decisions, online news consumption may also drive “echo chambers,” whereby individuals expose themselves only to information that conforms with their standing attitudes and beliefs. Our study aims to assess the impact of reliance on conservative and liberal online news sources on risk perception and mitigation behaviors associated with COVID-19. The results are discussed in terms of their theoretical implications for the study of online echo chambers and their practical implications for public health officials and emergency managers.

### Polarized COVID-19 Coverage

In the United States, science has a long history of being politicized. Analyzing public trust in science from 1974 to 2010, Gauchat^1^ found that individuals identifying as liberal or moderate had relatively stable attitudes toward science, whereas those who identified as conservative experienced decreased trust in science over time. The downward trend was long-term and sustained rather than being caused by a specific event or topic. The trend itself is alarming, but the implications are also dangerous, considering the role of scientists in fields such as medicine and public health. For example, extant research has suggested that vaccination attitudes, intentions, and behaviors differ between liberals and conservatives, such that more positive attitudes, stronger health behavior intentions, and higher vaccination rates are seen among liberals when compared with conservatives.^2-8^

More recently, research on the 2014 Ebola outbreak in the United States and the COVID-19 pandemic supports the ongoing political rift in perceptions of health and science. Adida et al ^9^ found that politicization of the Ebola crisis resulted in more exclusionary and negative attitudes toward immigrants among conservatives. The outcome variable was not directly related to public health, but this attitude change suggests implications beyond individual health behaviors when health topics are politicized.

Evidence suggests that the politicization of COVID-19 through media outlets and comments from prominent politicians is driving differences in how individuals of different political ideologies understand the crisis. In a content analysis of US newspapers and televised network news coverage from March to May 2020, researchers found that politicians appeared more frequently in newspaper coverage than scientists, but they were featured equally on broadcast news.^10^ This suggests that media coverage of COVID-19 devotes as much or more time to the perspectives of politicians as health experts. Given the public's reliance on mediated communication, especially early in the crisis, this finding is perhaps not surprising due to the polarization of COVID-19 information.^11^ This finding does not, however, suggest a direct effect of media coverage on audience members, but rather that the framing of COVID-19 likely has implications for how people make sense of the ongoing epidemic.

Another recent study showed that political conservatism inversely predicted compliance with social distancing, a mitigation behavior aimed at reducing the spread of COVID-19.^12^ Even after controlling for other demographic characteristics such as belief in science and COVID-19 anxiety, political affiliation remained a significant predictor of COVID-19 behavior. There is at least preliminary evidence to suggest that politicization of COVID-19 has a meaningful impact on both attitudes and behaviors; however, it is unclear at what stages individuals are exposed to the politicization—through their media consumption, local or national politicians, or interpersonal connections. Message effectiveness will likely rely on political ideology and how severe individuals perceive the outbreak to be. The impact of this polarization may be related to the level of exposure to information that does not align with existing attitudes and beliefs.

### Selective Exposure

A long history of research in the social sciences documents a fundamental desire to defend attitudes, beliefs, and behaviors in the face of information to the contrary.^13^ Individuals will, therefore, avoid information that challenges their attitudes, beliefs, and behaviors, and instead gravitate toward that which supports their existing positions.^14,15^ Some evidence, however, suggests that confirmation bias may be weakened by a motivation for accuracy.^16,17^ When the outcome of an information search is personally important or relevant to an individual, they may favor accuracy over confirmation of their beliefs, thus, weakening the echo-chamber effect. A recent meta-analysis of 91 studies spanning 52 years provides strong evidence for confirmation bias toward information that supports existing attitudes, beliefs, and behaviors, with accuracy motivation as a small but relevant moderator.^18^

When considered specifically in the context of partisan political content, ample evidence suggests that selective exposure to information confirming one's existing positions will serve to polarize them further.^19-23^ The evidence for a reverse relationship, whereby polarized opinions drive individuals to seek only congenial information, which supports preexisting beliefs and attitudes, has also received some support.^23,24^ This is consistent with Slater's^25^ arguments for a “spiral” effect, whereby continued exposure to partisan information compounds attitudinal polarization, as the motivating power of extreme attitudes on selective exposure grows over time. The plausibility of this nonlinear effect is even more evident when considering the range of content now available through interactive media, and, therefore, recent research has focused on these echo chambers driven by algorithms.

### Echo Chambers

The term “echo chamber” refers to a situation in which only certain ideas, beliefs, and sentiments are shared, such that those inside the echo chamber encounter only information they already agree with.^26,27^ Members of an echo chamber desire and distribute content “that both conforms to the norms of their group and tends to reinforce existing beliefs.”^28^ Those within the echo chamber, therefore, have little choice in opinion diversity, and those who disagree with the dominant positions of an echo chamber are considered wrong, misinformed, or malevolent.

Although echo chambers can be online or offline, the internet has enabled new opportunities for echo chambers to develop, namely through algorithmic filter bubbles.^28^ To help personalize our online experience, social media and search engine algorithms suggest content related to what we have already consumed on the basis that we will enjoy it more than disagreeable or unfamiliar content.^29^ The algorithms filter out incompatible information and create a bubble of like-minded resources.

Concepts explicated in the mass communication literature may contribute to the creation of echo chambers and opinion reinforcement through fragmentation, polarization, and homophily.^28^ Fragmentation is the process by which information that previously was accessible through only a handful of sources (eg, local newspaper, major newscasts) is now widely available among an expansive range of sources.^30^ Polarization “occurs when audiences diverge and are segmented based on an issue or interest.” ^28^ When audiences can choose from many information sources that present diverse opinions, the possibility of fragmentation and polarization increases, resulting in a viable environment for an echo chamber to develop.^28^ Finally, homophily refers to the tendency of individuals to interact with those who they believe share the same opinions and orientations.^31^ When individuals find alignment with others and their preexisting beliefs are reinforced in these segmented environments, they may become more dependent on such sources and turn to them for information, guidance, and behavioral advice.^32^

While some crisis and risk communication research mentions echo chambers, scholarship that explicitly examines echo chambers has been conducted primarily in the contexts of social media research and political communication. Results of these studies are famously mixed. Some researchers argue for the existence and impact of echo chambers with empirical research.^33,34^ Others argue that having unlimited access to opposing views and opinions weakens the impact of the homogeneous information shared within echo chambers and report little evidence of the impact of an echo chamber effect.^35,36^ Notably, research addressing echo chambers in the context of disease outbreaks or pandemics largely supports their existence. For example, research suggests that echo chambers impacted conversations about measles vaccination on Italian social media and the 2015 measles outbreak in California.^37,38^ Further, the impact of echo chambers is discussed frequently in health communication literature and related fields.^39,40^

Conflicting findings from research on echo chambers may be a result of how the research was conducted. A vast majority of echo chamber research has focused on a single platform, often Twitter and, in rare instances, Facebook.^28^ By focusing on only certain applications or social media specifically, there is much left to be desired in our understanding of the formation of and evidence for echo chambers. While examining web-driven content, our study looks beyond platforms to address general information-seeking behavior while focusing on the political perspective of the source. Furthermore, a long history of research in health, crisis, and risk communication has established a link between information seeking, risk perception, and resultant motivations and behaviors.^41-44^ Given the long-documented connection between information seeking and both risk estimation and risk mitigation, we propose the following hypotheses:

- Hypothesis 1: Information seeking positively predicts general risk perception
- Hypothesis 2: Information seeking positively predicts mitigation
- Hypothesis 3: Information seeking positively predicts risk estimation

Conflicting findings about echo chambers may complicate the relationships between information seeking and the 3 outcome variables assessed: general risk perception, mitigation, and risk estimation. Recent results suggest that echo chambers would logically affect the strength of the relationships—reliance on liberal-leaning news sources would magnify them and reliance on conservative news sources would weaken them. However, the literature is inconsistent in providing evidence to support the effect of echo chambers in regard to politicized content. To that end, we proposed the following research question: Are the relationships between information seeking and general risk perception, mitigation, and risk estimation moderated by a reliance on conservative or liberal websites/platforms?

## Methods

We distributed an online survey to a proportional, stratified sample of US citizens between April 21 and June 23, 2020. We collected data through Qualtrics online survey software (Qualtrics, Provo, UT). Respondents received $2.25 for their participation. We included a total of 5,019 surveys in our analysis after checking for completion and data quality—such as evidence of straightlining (clicking on the same response over and over) and irregular completion times.

The average respondent age was 45.6 (standard deviation [SD] = 17.8). In terms of sex, 2,435 (48.5%) respondents identified as male, 2,558 (51.1%) identified as female, 25 identified as other, and 1 provided no answer. People who identified as Caucasian comprised 61.5% of the sample, followed by Latinx (17.1%), African American (13.6%), Asian (6.0%), American Indian or Alaska Native (1.1%), Native Hawaiian or Pacific Islander (0.1%), and “other” (0.5%). Self-reports of income indicated 18.7% reported making less than $24,999 per year, 24.4% between $25,000 and $49,999, 19.4% between $50,000 and $74,999, 13.2% between $75,000 and $99,999, and 23.7% over $100,000. In regard to education level, 11.6% reported having less than a high school education, 29.5% a high school diploma, 16.6% some college, 9.8% an associate's degree, 20.5% a bachelor's degree, and 12% some kind of advanced degree. All 50 states, Washington, DC, and Puerto Rico were proportionately represented in the sample.

### Instrumentation

**Information seeking.** Survey questions (available at www.liebertpub.com/doi/suppl/10.1089/hs.2020.0176) used to assess information seeking were adopted from past research.^45^ Respondents estimated the number of hours spent seeking information about COVID-19 on a typical weekday, Saturday, and Sunday. This was converted to an estimate of information-seeking hours per week [(weekday × 5) + Saturday + Sunday)], (mean [M] = 42.88, SD = 44.59).

**General risk perception.** Participants were asked to complete the Event Hazard/Outrage Scale.^46^ The scale includes 32 items intended to measure generalized risk perceptions and negative emotional responses associated with them. Confirmatory factor analysis supported a 2-factor solution (confirmatory factor index [CFI] = 0.91, root mean square error of approximation [RMSEA] = 0.09). Only the hazard measure was examined in the current analyses (α = .91).

**Probability estimation.** To assess the estimation of specific risks, we used 3 survey questions adapted from previous research.^47^ Participants were asked to estimate the percentage of the US population that will become infected with COVID-19 (M = 48.47, SD = 26.50), the percentage of those infected who will develop a serious illness (M = 37.39, SD = 26.56), and the percentage of those infected who will die as a result of the disease (M = 30.80, SD = 28.46).

**Risk mitigation.** Participants were asked a series of yes/no questions as to whether they had engaged in each of 7 protective actions recommended by the Centers for Disease Control and Prevention (CDC)^48^; these included keeping a distance of 6 feet from others, avoiding touching one's face, using hand sanitizer, covering one's mouth when sneezing or coughing, washing hands more regularly, cleaning and disinfecting a home more frequently, and staying home from work or school. Positive responses were summed to produce a measure of mitigation actions (M = 6.11, SD = 1.36).

**Reliance on polarized websites.** In a series of items, participants were asked to evaluate their degree of dependency on specific websites for information about COVID-19, with instructions to consider their use of these sources across all media platforms. The response options included 25 different news outlets drawn from a broad range of political inclination and reliability (see adfontesmedia.com). Of these 25 sources, 6 websites were chosen from the extreme left (*Buzzfeed*, *Daily Kos, Huffington Post*, *Mother Jones*, *New Republic, Slate*) and the extreme right (*Blaze Media*, *Breitbart News Network*, *The Daily Caller*, *Infowars, Newsmax*, *One America News Network*) to be used as measures of reliance on polarized web content. Support was found for the hypothesized 2-factor model (RMSEA = 0.09, CFI = 0.97). Factor loadings ranged from 0.79 to 0.92. Reliability was found at α = .95 for reliance on liberal websites and α = .97 for reliance on conservative websites. Estimates of reliance on conversative and liberal websites were obtained for all respondents in the sample.

**Demographics.** Participants were asked a series of demographic questions, including age, sex, income, and ethnicity. For purposes of analysis, sex and ethnicity were both recoded into male/female and white/nonwhite.

## Results

Hierarchical regression was used to address the 3 hypotheses, and the moderating effects proposed in the research question were examined using the PROCESS macro for IBM SPSS Statistics for Windows version 25.0 (IBM Corp, Armonk, NY).^49^ For the regression analyses, demographic indicators were included in the first regression equation in order to control for extraneous variance; aggregate information seeking was then added, and the models were compared in terms of fit and variance. The research question was examined using a model that included information seeking as the predictor, with reliance on conservative websites and reliance on liberal websites as the moderators (PROCESS Model 2). For the moderation analyses, all variables were centered and converted to standard scores to produce standardized regression coefficients for both the main effects and interactions. Moderation—examining effects at low, mid, and high levels of a moderating variable—is then probed at the mean and 1 standard deviation above and below the mean for the moderators of interest.^49^

The results of the regression analysis largely failed to support Hypotheses 1 and 2: that information seeking would predict both general risk perception and mitigation. For general risk perception, the initial model was found to be statistically significant (*F*_4,4789_ = 29.65; *P <* .001; coefficient of determination [*R*^2^] = 0.02). The addition of information seeking to the model, while statistically significant, accounted for a trivial change in variance (*F*_1,4788_ = 14.48; *P <* .001; Δ*R*^2^ = 0.003; β = .06). By way of comparison, both women (β = .11; *P <* .001) and older respondents (β = .14; *P <* .001) expressed more general risk perception.

A similar pattern of results was detected in the findings for Hypothesis 2. For mitigation behaviors, the initial model was found to be statistically significant, while accounting for a small amount of variance (*F*_4,4592_ = 16.05; *P <* .001; *R*^2^ = 0.01). Adding information seeking to the model failed to significantly improve variance accounted for (*F*_1,4591_ = 14.48; *P* < .51) Slightly more mitigation behaviors were reported among women (β = .09; *P <* .001) and nonwhite (β = .06; *P <* .001) respondents.

Hypothesis 3 addressed the impact of information seeking on specific estimates of risk probability. Analyses for all 3 outcomes variables lend support to Hypothesis 3. For the estimate of the percentage of the US population that would become infected, the initial model was statistically significant (*F*_4,4780_ = 79.91; *P <* .001; *R*^2^ = 0.06). The addition of information seeking significantly improved the model (*F*_1,4779_ = 644.30; *P <* .001; Δ*R*^2^ = 0.17; β = .36). Again, women reported higher perceived infection rates (β = .15; *P <* .001), whereas older respondents (β = -.06; *P <* .001) and those of higher income (β = -.04; *P <* .006) reported slightly lower perceived infection rates.

Similar findings were detected for the estimation of the percentage of those infected who would develop serious health problems. The initial model was statistically significant (F_4,4768_ = 120.04; *P <* .001; *R*^2^ = 0.09). The addition of information seeking significantly improved the model (*F*_1,4767_ = 1765.93; *P <* .001; Δ*R*^2^ = 0.25; β = .53). Women (β = .10; *P <* .001) and nonwhite (β = .04; *P <* .006) respondents reported slightly higher estimates, whereas older (β = -.04; *P <* .001) and wealthier (β = -.10; *P <* .001) respondents reported slightly lower estimates. This pattern was similar for estimates of those infected who would die from the disease, with the initial model showing F_4,4769_ = 145.47; *P <* .001; *R*^2^ = 0.11. The addition of information seeking significantly improved the model (*F*_1,4768_ = 2333.81; *P <* .001; Δ*R*^2^ = 0.29; β = .58). Again, women (β = .08; *P <* .001) and nonwhite (β = .05; *P <* .001) respondents reported slightly higher estimated fatality rates, whereas older (β = -.06; *P <* .001) and wealthier (β = -.10; *P <* .001) respondents reported slightly lower estimates.

**Figure 1.**
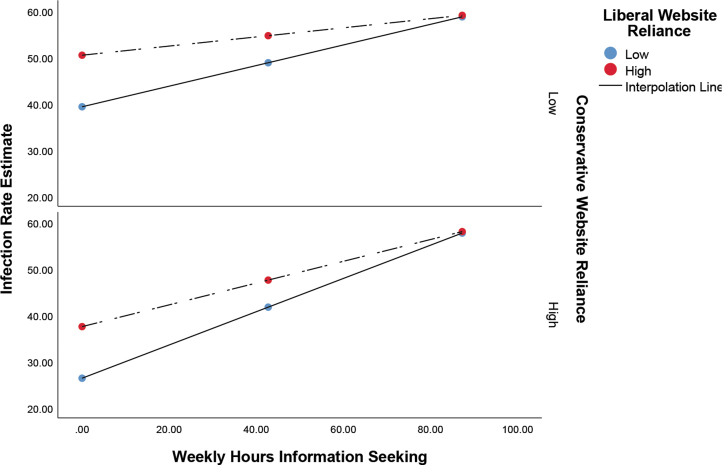
Effect of information seeking on infection rate estimates as moderated by reliance on liberal and conservative websites. The x-axis shows the estimated weekly number of hours spent seeking information about COVID-19.

Given the failure to support Hypotheses 1 and 2, subsequent moderation analyses probing the research question were performed only on the outcome variable associated with Hypothesis 3: risk estimation. In each case, the effect of information seeking on the outcome variable was examined, with reliance on liberal and conservative websites serving as moderators. For estimates of the infection rate in the population, a significant overall model was detected (*F*_5,4769_ = 167.18; *P <* .001; *R*^2^ = 0.15). The main effect for information seeking was found to be β = .38 (*P <* .001; 95% confidence interval [CI], 0.35 to 0.41), while the main effect for liberal websites was β = .11 (*P <* .001; 95% CI, 0.06 to 0.16), and for conservative websites was β = -.13 (*P <* .001; 95% CI, -0.19 to -0.08). Significant interaction effects were also detected between information seeking and liberal websites (β = -.10; *P <* .001; 95% CI, -0.15 to -0.05), and information seeking and conservative websites (β = .11; *P <* .001; 95% CI, 0.07 to 0.17). When probed at 1 standard deviation from the mean, the results suggest that high information seekers express similar estimates of infection rates, regardless of reliance on conservative or liberal websites. By way of comparison, among low information seekers, differences are clearly evident, with those reporting low reliance on liberal websites and high reliance on conservative websites indicating the lowest estimate of perceived risk (Figure 1). Our analysis suggests a stronger effect of information seeking on perceived risk of infection among those with a higher reliance on conservative websites (Table 1).

**Table 1.** Standardized Conditional Effects of Information Seeking on Infection Rate Estimates at Values of Reliance on Liberal and Conservative Websites

Liberal	Conservative	Effect	SE	t	P Value	95% CI
Low	Low	.3745	.0245	15.2892	<.001	0.3264-0.4225
Low	Middle	.4875	.0330	14.7598	<.001	0.4227-0.5522
Low	High	.6041	.0543	11.1181	<.001	0.4975-0.7106
Middle	Low	.2702	.0310	8.7213	<.001	0.2095-0.3309
Middle	Middle	.3832	.0164	23.3754	<.001	0.3511-0.4154
Middle	High	.4998	.0302	16.5649	<.001	0.4406-0.5590
High	Low	.1660	.0524	3.1644	.0016	0.0631-0.2688
High	Middle	.2790	.0296	9.4187	<.001	0.2209-0.3370
High	High	.3955	.0173	22.8606	<.001	0.3616-0.4295

Abbreviations: CI, confidence interval; SE, standard error; *t*, t test.

**Figure 2.**
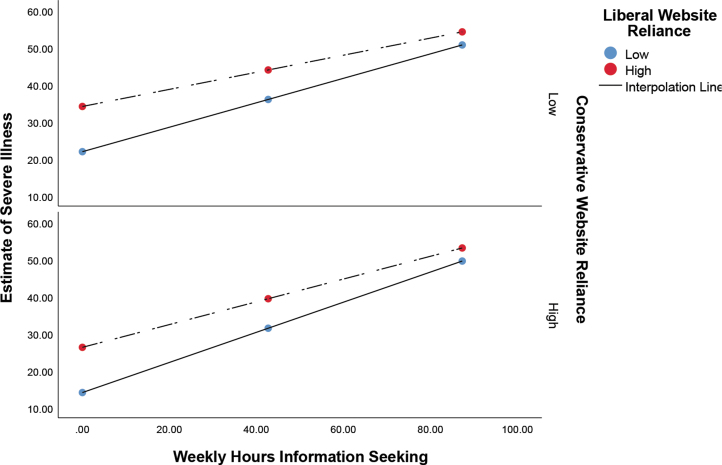
Effect of information seeking on estimates of severe illness as moderated by reliance on liberal and conservative websites. The x-axis shows the estimated weekly number of hours spent seeking information about COVID-19.

A nearly identical pattern emerges in the data for estimates of the number of those infected who will develop a serious illness. A significant overall model was detected at *F*_5,4756_ = 434.78 (*P <* .001; *R*^2^ = 0.32). The main effect for information seeking was found to be β = .53 (*P <* .0019; 95% CI, 0.51 to 0.56), while the main effect for liberal websites was β = .15 (*P <* .001; 95% CI, 0.10 to 0.20) and for conservative websites was β = -.09 (*P <* .001; 95% CI, -0.14 to -0.04). Significant interaction effects were again detected between information seeking and liberal websites (β = -.08; *P <* .001; 95% CI, -0.13 to -0.04), and information seeking and conservative websites (β = .07; *P <* .001; 95% CI, 0.02 to 0.11). Once again, high information seekers express similar estimates of those who will develop a serious illness. For low information seekers, again those reporting low reliance on liberal websites and high reliance on conservative websites indicated the lowest estimates (Figure 2, Table 2).

**Table 2.** Standardized Conditional Effects of Information Seeking on Estimates of Severe Illness at Values of Reliance on Liberal and Conservative Websites

Liberal	Conservative	Effect	SE	t	P Value	95% CI
Low	Low	.5541	.0221	25.1031	<.001	0.5108-0.5973
Low	Middle	.6173	.0298	20.7488	<.001	0.5590-0.6756
Low	High	.6826	.0489	13.9511	<.001	0.5867-0.7785
Middle	Low	.4707	.0279	16.8716	<.001	0.4160-0.5254
Middle	Middle	.5339	.0148	36.1420	<.001	0.5459-0.5629
Middle	High	.5992	.0272	22.0550	<.001	0.4406-0.6525
High	Low	.3873	.0472	8.2020	<.001	0.2947-0.4799
High	Middle	.4505	.0267	16.8892	<.001	0.3982-0.5028
High	High	.5158	.0156	33.1104	<.001	0.4853-0.5464

Abbreviations: CI, confidence interval; SE, standard error; *t*, t test.

**Figure 3.**
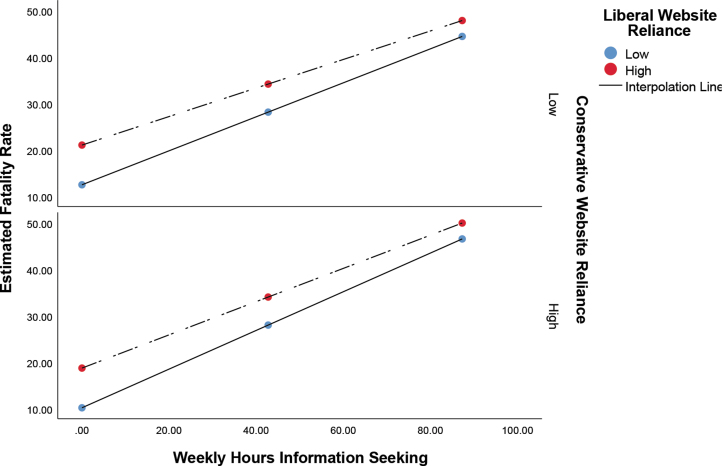
Effect of information seeking on fatality estimates as moderated by reliance on liberal and conservative websites. The x-axis shows the estimated weekly number of hours spent seeking information about COVID-19.

For the outcome addressing estimates of those infected who will die as a result of COVID-19, a slightly different pattern emerges. The overall model was found significant (*F*_5,4760_ = 582.42; *P <* .001, *R*^2^ = 0.38). A significant main effect was detected for information seeking (β = .57; *P <* .001; 95% CI, 0.54 to 0.59), reliance on liberal websites (β = .11; *P <* .001; 95% CI, 0.06 to 0.15), and the interaction effect between them (β = -.05; *P <* .05; 95% CI, -0.09 to -0.01. No significant main effect was detected for reliance on conservative websites, nor was an interaction effect detected between information seeking and reliance on conservative websites (Figure 3). While subtle, the conditional effects suggest a stronger effect of information seeking on fatality rate among those less reliant on liberal websites (Table 3).

**Table 3.** Standardized Conditional Effects of Information Seeking on Fatality Estimates at Values of Reliance on Liberal and Conservative Websites

Liberal	Conservative	Effect	SE	t	P Value	95% CI
Low	Low	.5736	.0210	27.2825	<.001	0.5324-0.6149
Low	Middle	.6127	.0284	21.6097	<.001	0.5571-0.6683
Low	High	.6530	.0466	13.9994	<.001	0.5615-0.7444
Middle	Low	.5276	.0266	19.8406	<.001	0.4755-0.5797
Middle	Middle	.5666	.0141	40.2509	<.001	0.5390-0.5942
Middle	High	.6069	.0259	23.4240	<.001	0.5561-0.6577
High	Low	.4816	.0450	10.6988	<.001	0.3933-0.5698
High	Middle	.5206	.0254	20.4770	<.001	0.4708-0.5705
High	High	.5609	.0149	37.7244	<.001	0.5317-0.5900

Abbreviations: CI, confidence interval; SE, standard error; *t*, t test.

## Discussion

The findings for Hypothesis 1—that information seeking does not motivate general risk perception—were somewhat unexpected given a lengthy history of research connecting information seeking to perceptions of risk severity. The findings for Hypothesis 2—that information seeking does not motivate mitigation—are also puzzling given a long history of research connecting risk information to protective action. This may be a product of the relative simplicity of CDC guidelines, and the fact that suggestions like wearing a facemask and washing one's hands do not require a great deal of effort. While CDC guidelines have shifted over the course of the pandemic, the individual-level recommendations captured here were fairly consistent during the time data was collected (April through June 2020). The mean across this outcome variable was fairly high for the entire sample (M* =* 6.11; SD = 1.36 on a 7-point scale), suggesting there may simply have been little variance in the outcome. Hypothesis 3—that information seeking would predict specific estimates of risk—was supported by our analysis. This can perhaps be traced to the underlying processes surrounding information seeking through websites. Whether seeking information from conservative or liberal sources, information seeking requires some degree of active processing. It may be that heavy information seekers, by definition, engage in active processing and are, therefore, better able to encode risks and process them into specific estimates of infection, health risk, and mortality.

Findings for the research question—concerning the moderating effect of reliance on conservative and liberal websites—may shed further light on the findings for all 3 hypotheses and are the most interesting and impactful findings in this study. With regard to echo chambers, our findings for the research question largely indicate that higher information seekers did not experience attitudinal polarization; in fact, across all 3 outcome variables the risk estimates for those reliant on liberal and conservative news content converged at higher levels of information seeking. In other words, lower information seekers, those reliant on conservative sources, reported the lowest levels of risk probability, whereas those reliant on liberal sources reported the highest (Figures 1, 2, and 3). At high levels of information seeking these differences disappear. Accordingly, the impact of information seeking on risk estimates is higher among those reliant on conservative websites, since they have further to go to converge; this is evident in the standardized conditional effects (Tables 1, 2, and 3).

In short, these findings run counter to the notion of echo chambers, and more closely approximate the argument of Messing and Westwood^35^—that those who engage in high levels of information seeking likely gather information from a range of sources. It may also be the case that high information seekers draw from a range of platforms and may be more open to information that does not align with (or challenges) existing attitudes and beliefs. This would also explain the failure to find a relationship between information seeking and both general risk perception and mitigation (Hypotheses 1 and 2). If high information seekers draw from liberal and conservative news sources, they would likely be exposed to or open to a range of perspectives, including those suggesting high risk and the need to take protective action. This exposure could potentially weaken direct effects between overall information seeking and the variable outcomes.

If politicized underreporting of the threats associated with COVID-19 is a concern, the lower information seekers may be more at risk, as this is where clear differences are evident in risk estimation by source preference. This finding is particularly alarming when considering Slater's^25^ arguments concerning polarization spirals; if low information seekers with polarized conservative opinions consume congenial information about the pandemic, and only congenial information, they may be likely to double down on their positions concerning specific risk estimates and become even more inclined to seek information that affirms those positions.

Although this is a single study in a highly specified context, health officials may wish to consider these findings when countering misinformation and understatements of risk. The most impressionable audiences may be those who seek the least amount of information and are, therefore, susceptible to information that confirms their biases. Identifying and segmenting these audiences along media preferences and demographic and social strata may enable health officials to target risk messages to those least likely to actively seek information.

## Supplementary Material

Supplemental data
