# Supplementary material for: COVID-19 Echo Chambers: Examining the Impact of Conservative and Liberal News Sources on Risk Perception and Response
Source: Health Secur. 2021 Feb 18;19(1):21–30. doi: 10.1089/hs.2020.0176 (PMC9195485; doi:10.1089/hs.2020.0176)
Supplement: Supplemental data [file Supp_Data.docx]

**Addendum: Survey Instrumentation**

Information seeking (open ended):

Please think about the sources you indicated as being important to you. How many hours do you spend seeking information related to the COVID-19 pandemic......

              1. On a typical weekday?

              2. On a typical Saturday?

              3. On a typical Sunday?

General Risk Perception:

Scoring: 1= Strongly disagree 2= Disagree 3= Somewhat disagree 4= Neither agree nor disagree

5= Somewhat agree 6= Agree 7= Strongly agree

The following items are descriptions of varying responses to information about the COVID-19 pandemic. Please tell us how you felt when you found out about the pandemic:

It looked as though people were going to be hurt.

I was afraid people would be emotionally affected

Losing my personal assets was a concern

I felt COVID-19 could cause emotional harm to a friend or loved one

I figured it wasn’t likely to hurt anyone

I feared for the life of a loved one

I saw no reason why COVID-19 would destroy anything that belonged to me

I felt as though my life was in jeopardy

I felt COVID-19 could threaten the lives of others

I felt COVID-19 could cause me emotional harm

It looked like COVID-19 would shake up a lot of people

It looked as though COVID-19 would disrupt my community

I feared COVID-19 would lead to a reduction in my quality of life

I was concerned that a lot of people would suffer

I felt that I might experience monetary loss as a result of COVID-19

It looked as though COVID-19 might destroy my community

Probability Estimation (open ended):

Given your understanding of COVID-19, please answer the following as best as you can.

What percentage of the U.S. population is likely to become infected with COVID-19?

What percentage of those infected in the U.S. will develop a serious illness?

What percentage of those infected in the U.S. will die due to symptoms associated with the virus?

Mitigation:

Scoring: yes = 1 no = 0

Please indicate which of the following you have done to protect yourself from the COVID-19 virus:

Washed your hands more regularly

Used hand sanitizer

Taken extra care to avoid touching your face

Tried to keep a distance of at least 6 ft from others

Stayed home from work/school

Covered mouth when sneezing or coughing

Cleaned and disinfected your home more

Reliance on Polarized Web Sources:

Scoring: 1= not at all reliant 2= used rarely 3= used occasionally 4 = neutral 5= used frequently

6= used often 7= very reliant

We would like to ask you some questions about your reliance on specific news outlets for information about COVID-19. Please think about all the ways you have gathered information (TV, websites, mobile applications, etc.) and answer the following. How reliant have you been on the following sources?

Slate

Mother Jones

Huffington Post

BuzzFeed

New Republic

Daily Kos

Daily Caller

Newsmax

Breitbart

OANN

The Blaze

Infowars

Demographics:

Please indicate your age (open ended)

What is your sex?

1= male

2= female

3 = other

What is your primary ethnicity?

1 =White

2= Black or African American

7= Hispanic/Latinx

3= American Indian or Alaska Native

4= Asian

5= Native Hawaiian or Pacific Islander

6= Other

What is your household income?

1= Less than $24,999

2= 25,000 to 49,999

3= 50,000 to 74,999

4 =75,000 to 99,999

5 =$100,000 or more

6 =Prefer not to answer
